# METTL14 benefits the mesenchymal stem cells in patients with steroid-associated osteonecrosis of the femoral head by regulating the m6A level of PTPN6

**DOI:** 10.18632/aging.203778

**Published:** 2021-12-15

**Authors:** Cheng Cheng, Haoping Zhang, Jia Zheng, Yi Jin, Donghui Wang, Zhipeng Dai

**Affiliations:** 1 Department of Orthopedics, Henan Provincial People′s Hospital, Zhengzhou, Henan, China; 2 Department of Mini-invasive Spinal Surgery, Third Hospital of Henan Province, Zhengzhou, Henan, China

**Keywords:** osteonecrosis of the femoral head, mesenchymal stem cell, m6A, METTL14, Wnt signaling pathway

## Abstract

Imbalanced osteogenic/adipogenic differentiation of bone marrow mesenchymal stem cells (BMSCs) is considered the core pathological characteristic of steroid-associated osteonecrosis of the femoral head (SONFH). N6-Methyladenosine (m6A) is the most common type of RNA modification in eukaryotic cells and participates in various physiological and pathological processes. However, the relationship between m6A modification and SONFH has not been reported. In the present study, we aimed to explore the roles of m6A modifications and methyltransferase METTL14 in SONFH. Our results showed that the m6A levels were down-regulated in femoral head tissues and BMSCs from SONFH patients, and this effect was attributed to the reduction of METTL14. Furthermore, METTL14 overexpression in BMSCs from SONFH patients enhanced cell proliferation and osteogenic differentiation. We further identified PTPN6 as the downstream target of METTL14 by mRNA sequencing. Mechanistically, METTL14 regulated PTPN6 expression by increasing PTPN6 mRNA stability in an m6A-dependent manner. Moreover, PTPN6 knockdown abrogated the beneficial effects of METTL14 overexpression on BMSCs. Additionally, we found that METTL14 activated the Wnt signaling pathway, and this effect was caused by the interaction of PTPN6 and GSK-3β. In conclusion, we elucidated the functional roles of METTL14 and m6A methylation in SONFH BMSCs and identified a novel RNA regulatory mechanism, providing a potential therapeutic target for SONFH.

## INTRODUCTION

Steroid-associated osteonecrosis of the femoral head (SONFH) is a severe clinical complication caused by the use of glucocorticoids. Studies have confirmed that the occurrence and progression of SONFH are related to the course and dose of glucocorticoids [1]. With the increasing incidence of SONFH, the molecular mechanism urgently needs to be identified.

Bone marrow mesenchymal stem cells (BMSCs) are pluripotent stem cells with self-renewal and multidirectional differentiation potential and can differentiate into osteoblasts, adipocytes, endotheliocytes and chondrocytes [2–5]. Thus, BMSCs play a crucial role in bone metabolism, tissue repair and are considered ideal seed cells for tissue engineering [6–8]. Moreover, these cells are also closely related to the development of bone disorders. Recent studies have demonstrated that the progression of SONFH is associated with BMSCs dysfunction, which is mainly manifested by the defection of osteogenic differentiation and the enhancement of adipogenic differentiation [9–11]. In previous studies, BMSCs in proximal femoral bone marrow were significantly reduced in the rabbit model of SONFH, and BMSCs from SONFH patients had decreased proliferation and osteogenic activity [12–14]. Therefore, strategies promoting the BMSC proliferation and osteogenic ability may be a promising therapeutic method for SONFH.

RNA methylation accounts for more than 60% of RNA modifications [15]. Among them, m6A is the most common RNA methylation modification in eukaryotic cells [16]. In mammalian cells, the m6A modification is a reversible process regulated by m6A writers, erasers and readers [17]. The formation of m6A methylation is catalyzed by the methyltransferase complex that consists of m6A writers, among which METTL3, METTL14 and WTAP play vital roles [18]. Conversely, ALKBH5 and FTO act as erasers to reverse the m6A modification [19]. The readers consist of YTH domain proteins and the nuclear heterogeneous protein family, which are responsible for reading the methylation information and participating in RNA translation and degradation [20]. Previous studies have demonstrated that m6A modification involves in the life cycle of RNA, affecting the RNA stability, translation efficiency, alternative splicing and localization [21–24]. Aberrant levels of m6A have been confirmed to contribute to a variety of diseases, such as tumors, neurological diseases, and metabolic diseases [25–27], and m6A RNA methylation also regulates the fate of BMSCs [28] and involved in the progression of diseases related to BMSCs abnormalities. For instance, Liu et al. discovered that METTL3 promoted BMSCs osteogenesis by mediating m6A methylation of BMP2 transcripts and supposed that METTL3 could mitigate ovariectomy-induced osteoporosis [29]. Xie et al. revealed that TNF-α induced m6A modification in ELMO1 3′UTR triggers directional migration of mesenchymal stem cell in ankylosing spondylitis patients [30]. Wang et al. elucidated a new pathogenesis of osteoporosis, that is, FTO mediates Runx2 mRNA demethylation to inhibit the osteogenic differentiation of BMSCs [31]. However, the roles of m6A modification and the underlying regulatory mechanisms in SONFH remain unclear.

PTPN6 belongs to the protein tyrosine phosphatase family, which is mainly expressed in hematopoietic stem cells. PTPN6 regulates receptor tyrosine kinases by binding to target proteins and dephosphorylating phosphorylated tyrosine substrates, which play a significant role in cell differentiation, proliferation, and biological functions [32]. Previous studies have confirmed that PTPN6 is involved in the molecular mechanisms of the progression of various disease [33, 34]. However, the underlying mechanism by which PTPN6 is regulated, especially via m6A modification, and the roles of PTPN6 in SONFH are still unknown.

Here, we found that the levels of m6A modification and METTL14 expression in SONFH tissues and BMSCs were significantly lower than those in normal tissues and cells. Overexpression of METTL14 in SONFH BMSCs significantly enhanced cell proliferation and osteogenic differentiation, and regulated the expression of lineage-specific transcription factors including RUNX2, PPARγ and C/EBPs. Molecular mechanism analysis indicated that METTL14 mediated the m6A modification and stability of PTPN6 mRNA, which further up-regulated PTPN6 expression in SONFH BMSCs. Rescue experiments showed that PTPN6 knockdown abrogated the effects of METTL14 on cell proliferation and differentiation. Furthermore, we demonstrated that PTPN6-mediated METTL14 activated the Wnt signaling pathway by interacting with GSK-3β. Our study laid the foundation for future research on the roles of m6A modification in SONFH, and provided a novel target for the treatment of SONFH.

## RESULTS

### Identification of BMSCs

**Figure 1:**
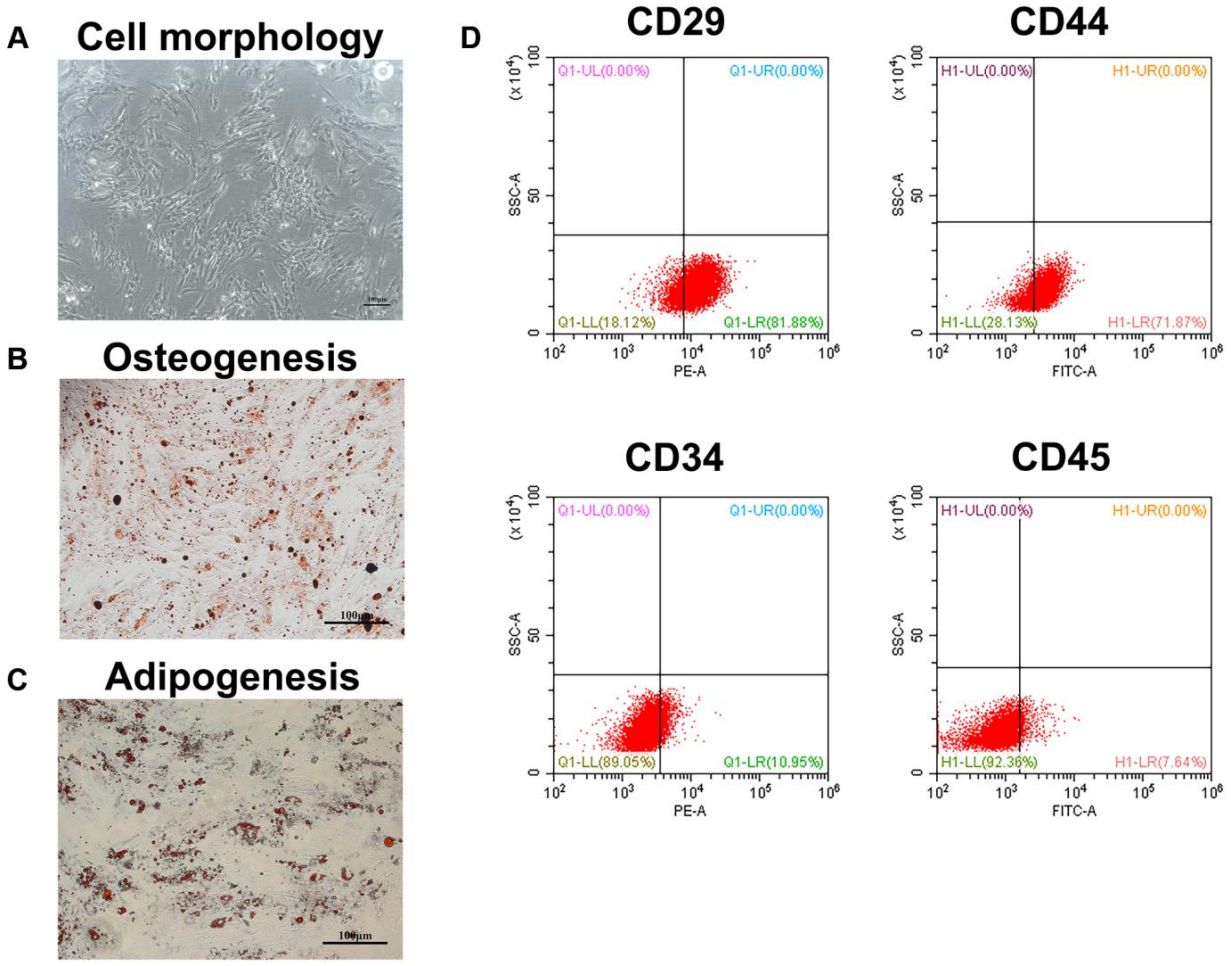
**Identification of BMSCs.** (**A**) The appearance of BMSCs cultured to the second generation. Scale bar = 100 μm. (**B**) The osteogenesis differentiation of BMSCs was confirmed by alizarin red staining. Scale bar = 100 μm. (**C**) The potential of BMSCs to differentiate into adipocytes was verified by oil red staining. Scale bar = 100 μm. (**D**) The surface markers of BMSCs were identified by flow cytometry.

When the cells were passaged to the second generation, they had a typical long fusiform appearance under the microscope (Figure 1A). Besides, the cells have the potential to differentiate into osteoblasts and adipocytes (Figure 1B, 1C). Furthermore, we analyzed four known surface markers associated with human bone marrow mesenchymal stem cells by flow cytometry. The results showed that the cells were positive for CD29 and CD44, but negative for CD34 and CD45 (Figure 1D). These results indicated that the isolated cells were human BMSCs.

### METTL14 was responsible for the aberrant m6A modifications in BMSCs from SONFH patients

**Figure 2:**
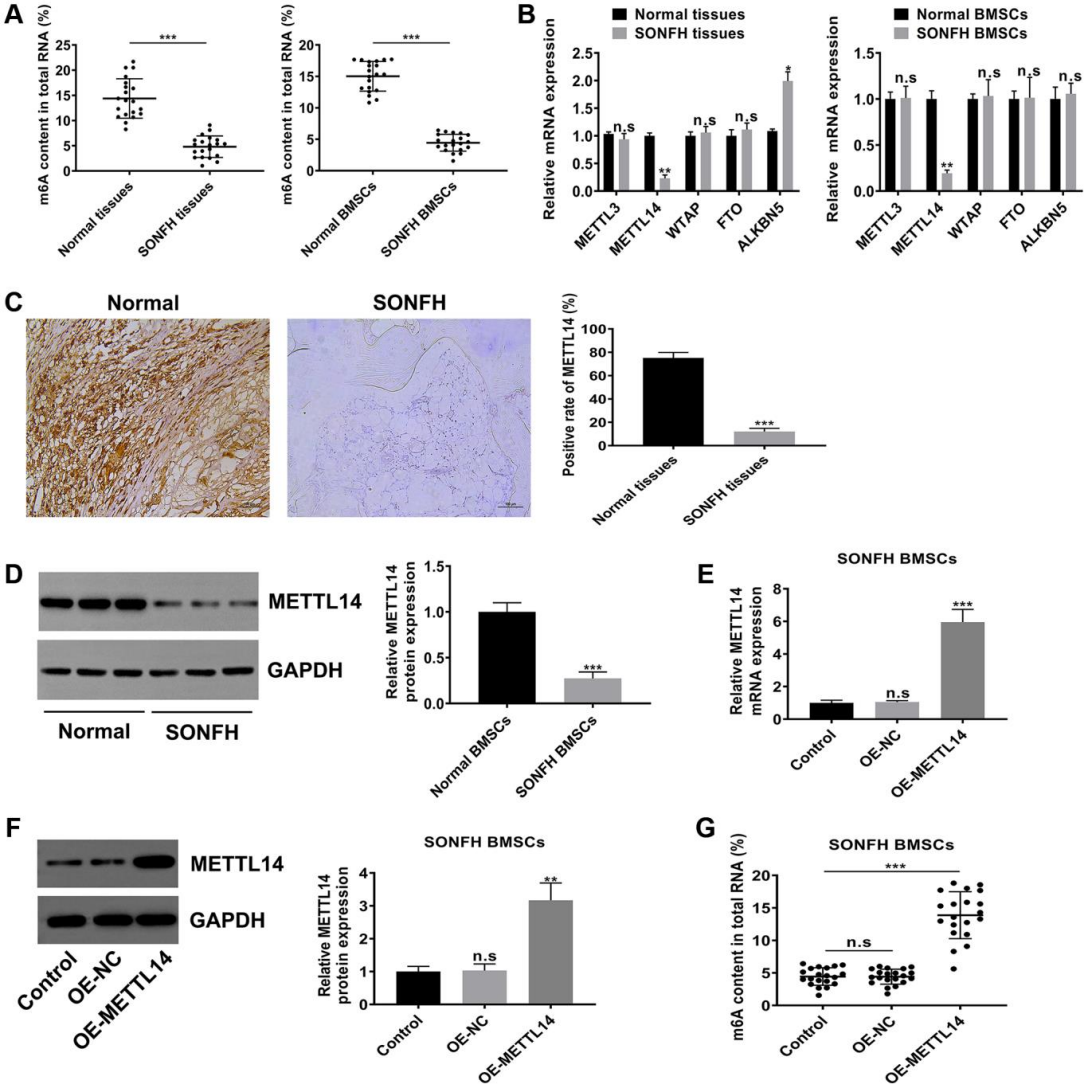
**METTL14 was responsible for the aberrant m6A modification in BMSCs from SONFH patients.** (**A**) The m6A content in total RNA of SONFH tissues and BMSCs (*n* = 20) and normal tissues and BMSCs (*n* = 20). (**B**) mRNA levels of m6A modification associated genes in SONFH tissues and BMSCs and normal tissues and BMSCs. (**C**) IHC assay determined the expression of METTL14 in osteonecrosis tissues and normal tissues. Scale bar = 100 μm. (**D**) The protein levels of METTL14 in SONFH BMSCs and normal BMSCs were measured by western blot. (**E**, **F**) The efficiency of METTL14 overexpression in SONFH BMSCs was confirmed by qRT-PCR and western blot analysis. (**G**) Quantitative m6A methylation assay was used to define the effect of METTL14 overexpression on the level of m6A modification in BMSCs from SONFH patients. ^*^P < 0.05, ^**^P < 0.01, ^***^P < 0.001, n.s is no significance.

To explore the potential role of m6A modification in SONFH, we initially examined the level of m6A in the total RNA of femoral head tissues and BMSCs. As shown in Figure 2A, the m6A content in SONFH tissues and BMSCs was significantly lower than that in normal group. According to the previous studies, m6A modifications are catalyzed by m6A methyltransferase and m6A demethylase, so we next examined the mRNA levels of m6A modification associated genes, including METTL3, METTL14, WTAP, FTO and ALKBH5. Interestingly, the m6A methyltransferase METTL14 was obviously down-regulated in osteonecrosis tissues and BMSCs from SONFH patients, while the demethylase ALKBH5 was up-regulated in SONFH tissues, but not in SONFH BMSCs. The levels of METTL3, WTAP and FTO exhibited no significant differences between the two groups (Figure 2B). Thus, we hypothesized that METTL14 was a critical regulator that contributed to the abnormal m6A modifications. We further performed IHC analysis with femoral head tissues, and found that the positive rate of METTL14 in necrotic femoral head tissues was significantly lower than that in healthy femoral head tissues (Figure 2C). Moreover, western blotting indicated that protein levels of METTL14 were down-regulated in BMSCs from SONFH patients compared to those in normal BMSCs (Figure 2D). To further verify the effect of METTL14 on the m6A modification in BMSCs, we overexpressed METTL14 in SONFH BMSCs, and qRT-PCR and western blotting were used to determine the transfection effect (Figure 2E, 2F). Then, we detected the m6A content in cells. The results showed that the m6A level was increased significantly upon METTL14 overexpression (Figure 2G). Together, these data demonstrated that the m6A level was decreased in SONFH femoral head tissues and BMSCs, and that METTL14 was the regulator involving the abnormal m6A modification.

### METTL14 up-regulation promoted the proliferation and osteogenesis of SONFH BMSCs

**Figure 3:**
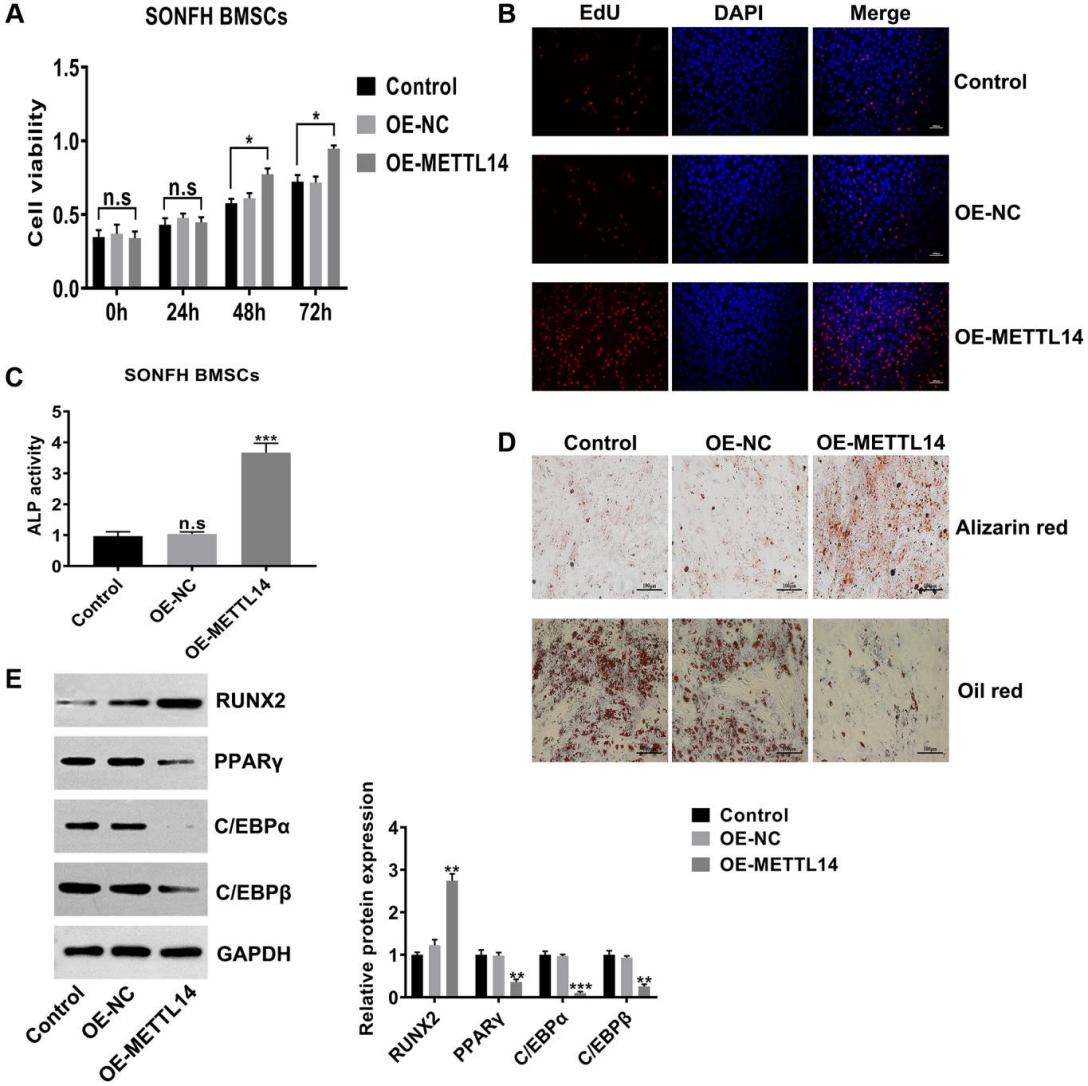
**METTL14 up-regulation promoted the proliferation and osteogenesis of SONFH BMSCs.** (**A**) The effect of METTL14 up-regulation on the cell viability of SONFH BMSCs was analyzed by CCK-8 assay. (**B**) EdU assay was used to detect the influence of METTL14 overexpression on the proliferation of SONFH BMSCs. Scale bar = 100 μm. (**C**) The ALP activity assay provided evidence that METTL14 up-regulation enhanced the ability of osteogenic differentiation of BMSCs from SONFH patients. (**D**) METTL14 overexpression promoted the ability of osteogenic differentiation and suppressed the adipogenesis capacity of SONFH BMSCs were confirmed by alizarin red staining and oil red staining. Scale bar = 100 μm. (**E**) The expression of osteogenic-related and adipogenic-related genes in BMSCs with METTL14 overexpression were analyzed by western blot. ^*^P < 0.05, ^**^P < 0.01, ^***^P < 0.001, n.s is no significance.

To investigate the biological functions of METTL14, we assessed the proliferation and osteogenesis abilities in SONFH BMSCs transfected with OE-METTL14. The results of CCK-8 and EdU assays confirmed that METTL14 overexpression significantly promoted the proliferation capacity of BMSCs (Figure 3A, 3B). Furthermore, METTL14 up-regulation augmented cell ALP activity (Figure 3C). Alizarin red staining and oil red staining showed that the ability to differentiate into osteoblasts was enhanced and the potential to differentiate into adipocytes was decreased when METTL14 expression was overexpressed (Figure 3D). Western blotting results demonstrated that with METTL14 overexpression, the levels of the osteogenic-related gene RUNX2 was up-regulated, while the expression of adipogenic-related genes PPARγ, C/EBPα and C/EBPβ were down-regulated in SONFH BMSCs (Figure 3E). Taken together, these results suggested that METTL14 promotes the proliferation and osteogenesis of BMSCs from SONFH patients.

### METTL14-dependent m6A modifications regulated the expression of PTPN6

**Figure 4:**
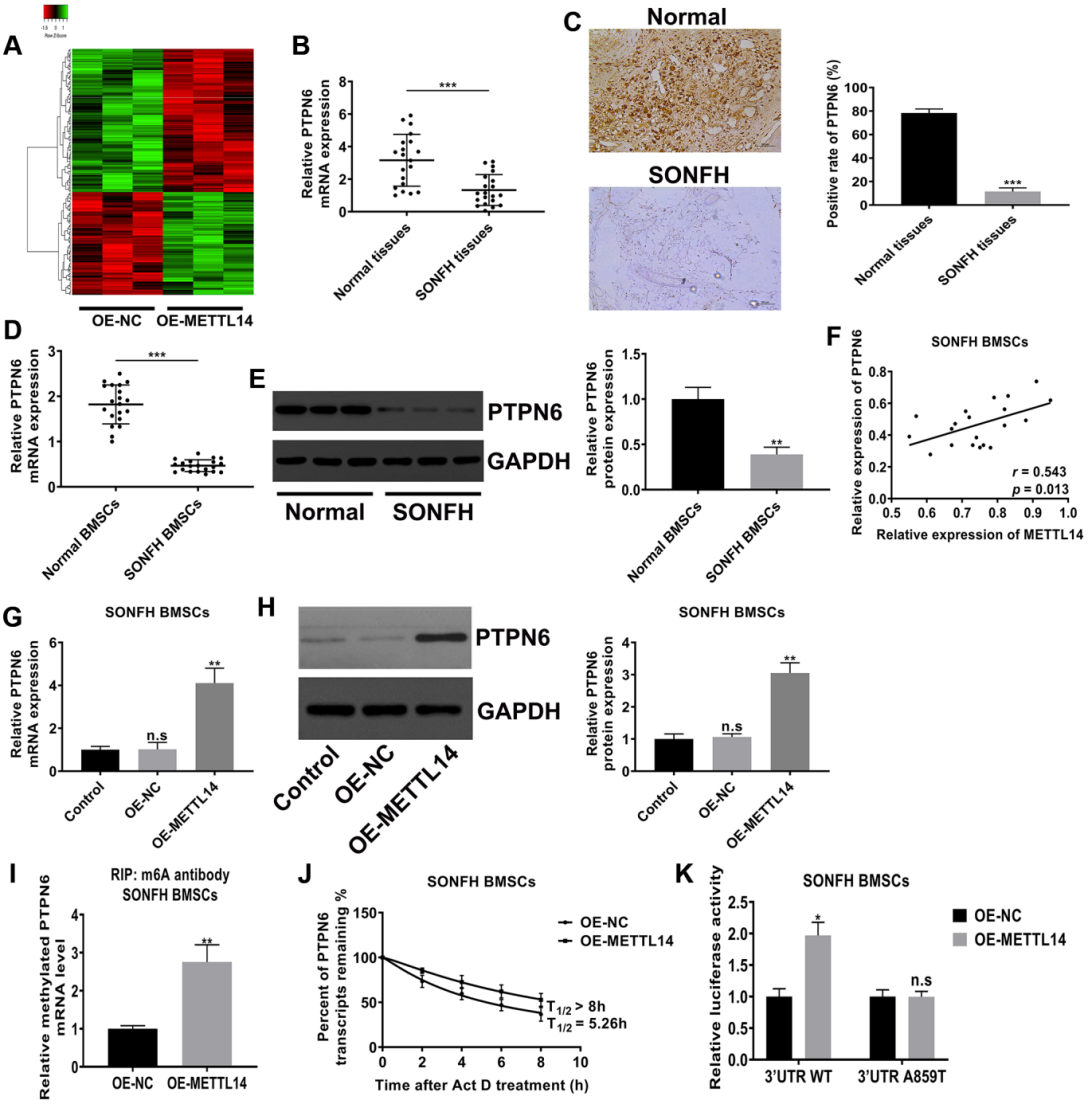
**METTL14-dependent m6A modification regulated the expression of PTPN6.** (**A**) Heat map of normalized gene expression levels of SONFH BMSCs transfected with OE-NC or OE-METTL14. Red indicates down-regulation of gene expression and Green indicates up-regulation of gene expression. (**B**) PTPN6 mRNA levels in normal tissues (*n* = 20) and osteonecrosis tissues (*n* = 20) were measured by qRT-PCR. (**C**) IHC assay detected the expression of PTPN6 in femoral head tissues of two group. Scale bar = 100 μm. (**D**, **E**) qRT-PCR and western blot quantified the abundance of PTPN6 in normal BMSCs and SONFH BMSCs. (**F**) The correlation between METTL14 and PTPN6 expression in SONFH BMSCs was analyzed by Pearson’s Correlation Coefficient. (**G**, **H**) qRT-PCR and western blot confirmed that PTPN6 expression increased with the up-regulation of METTL14 in SONFH BMSCs. (**I**) The methylated PTPN6 mRNA level in the METTL14 overexpressed BMSCs was determined by MeRIP-qPCR. (**J**) The mRNA half-life of PTPN6 in SONFH BMSCs transfected with OE-NC or OE-METTL14. (**K**) SONFH BMSCs were pre-transfected with wild-type or mutated PTPN6-3′UTR plasmids, and then treated with OE-NC or OE-METTL14. The luciferase activity was normalized to firefly luciferase activity. ^*^P < 0.05, ^**^P < 0.01, ^***^P < 0.001, n.s is no significance.

M6A modification mostly exists in mRNA and assumes as a form of posttranscriptional regulation, which further affects the expression of the modified gene. To identify the downstream target of METTL14, we performed mRNA sequencing on BMSCs from SONFH patients transfected with OE-METTL14 or OE-NC. Differentially expressed genes were screened based on *p*-value <0.05 and false discovery rate <0.05. Compared to control cells, METTL14-overexpressing cells had 101 genes with increased expression and 142 genes were decreased, and these genes were clustered analyzed and presented as a heat map (Figure 4A). Among differentially expressed genes, the expression of PTPN6 was most significantly affected by METTL14 (fold change was the highest). The data of qRT-PCR displayed that PTPN6 mRNA levels in osteonecrosis tissues was decreased compared with control femoral head tissues (Figure 4B). IHC assay also provided consistent results, the positive staining of PTPN6 in necrotic femoral head tissues was significantly less than that in normal tissues (Figure 4C). Moreover, the qRT-PCR and western blot results indicated that the level of PTPN6 in BMSCs of SONFH was significantly lower than that in normal BMSCs (Figure 4D, 4E), and PTPN6 mRNA expression was positively correlated with METTL14 mRNA in SONFH BMSCs (Figure 4F). Besides, in SONFH BMSCs, the mRNA and protein levels of PTPN6 were increased with METTL14 up-regulation (Figure 4G, 4H). We then investigated the m6A modification status of PTPN6 by performing MeRIP-qPCR assay. The results validated that the up-regulation of METTL14 enriched the m6A modification level of PTPN6 mRNA (Figure 4I). Furthermore, we carried out the RNA stability assay to analyze the half-life of PTPN6 mRNA. As shown in Figure 4J, the half-life of PTPN6 mRNA was 5.26 hours in SONFH BMSCs, while the half-life of PTPN6 was longer than 8 hours in METTL14 overexpression cells. To further elucidate the mechanism of METTL14 regulation of PTPN6 mRNA stability, we performed the luciferase reporter assay and found that METTL14 overexpression reinforced the activity of the luciferase construct containing the wild type 3′UTR of PTPN6 mRNA. Mutation at position 859 (A to T) almost completely rendered resistance to the luciferase activity of METTL14 overexpression (Figure 4K). Collectively, METTL14-dependent m6A modification regulates the expression of PTPN6 by enhancing the stability of PTPN6 mRNA.

### PTPN6 knockdown abrogated the beneficial effects of METTL14 overexpression on SONFH BMSCs

**Figure 5:**
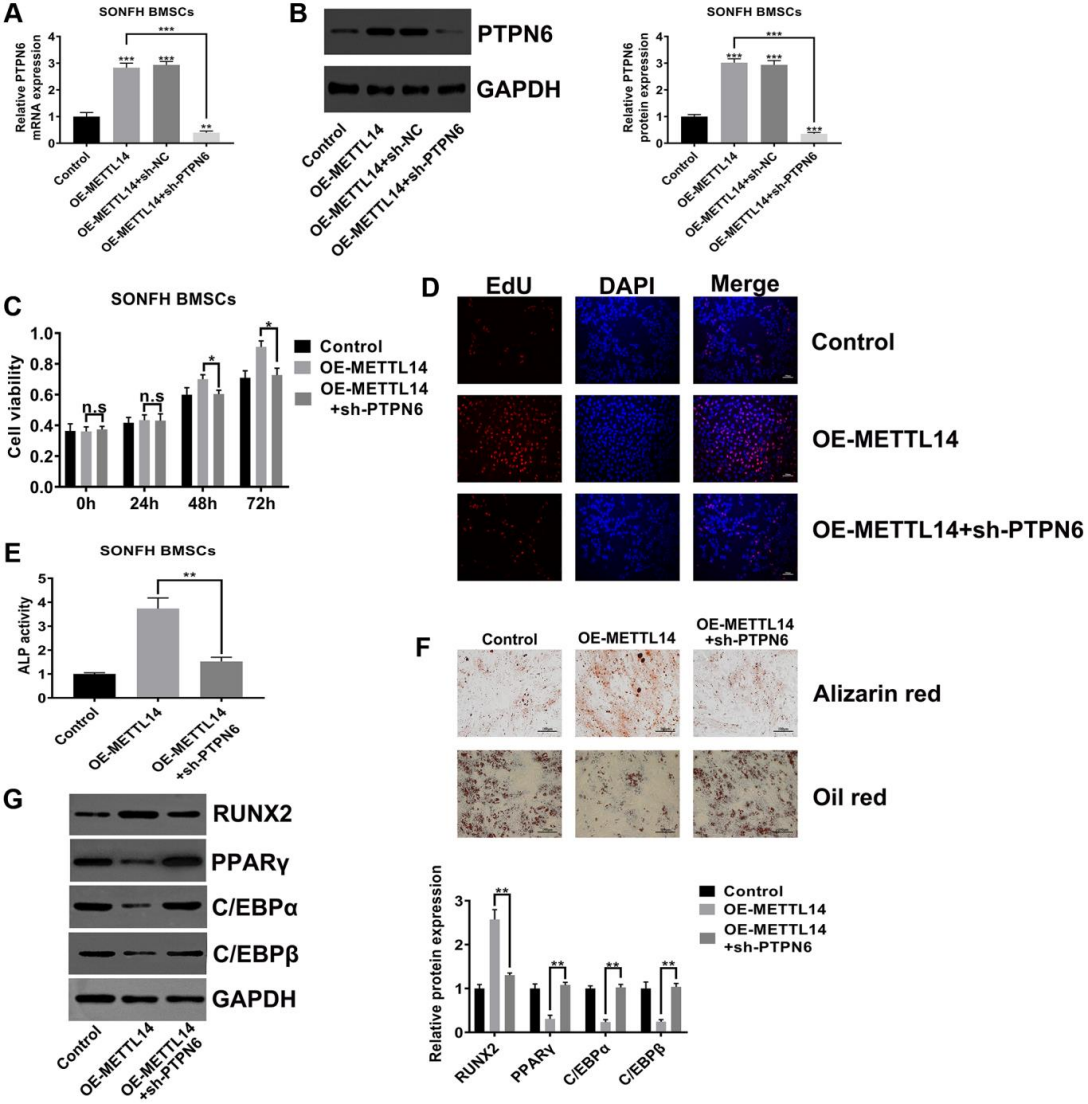
**PTPN6 knockdown abrogated the beneficial effects of METTL14 overexpression on SONFH BMSCs.** (**A**, **B**) qRT-PCR and western blot validated the efficiency of PTPN6 knockdown in METTL14 overexpression cells. (**C**, **D**) The CCK-8 and EdU assay evaluated the reversal effect of PTPN6 knockdown on cell proliferation induced by METTL14 overexpression. Scale bar = 100 μm. (**E**) The ALP activity assay was used to assess the reversal effect of PTPN6 knockdown on the osteogenesis promoted by METTL14 up-regulation. (**F**) The effects of sh-PTPN6 on the abilities of osteogenic differentiation and adipogenic differentiation of METTL14 overexpression cells were analyzed by Alizarin red staining and oil red staining. Scale bar = 100 μm. (**G**) Knockdown of PTPN6 counteracted the regulatory effect of METTL14 overexpression on osteogenic and adipogenic related genes. ^*^P < 0.05, ^**^P < 0.01, ^***^P < 0.001, n.s is no significance.

To explore whether PTPN6 is critical to the effects of METTL14 on SONFH BMSCs, we silenced PTPN6 in METTL14 overexpression cells. The efficiency of PTPN6 knockdown was confirmed by qRT-PCR and western blot assays (Figure 5A, 5B). The CCK-8 assay and EdU assay indicated that silenced PTPN6 dramatically abrogated the effect of METTL14 on cell proliferation (Figure 5C, 5D). The results of alizarin red staining, oil red staining and ALP activity assays demonstrated that PTPN6 knockdown reversed the promotion of METTL14 overexpression on the osteogenic differentiation of BMSCs (Figure 5E, 5F). In addition, when we weakened PTPN6 expression in METTL14-overexpressing cells, the protein level of RUNX2 was down-regulated, and those of PPARγ, C/EBPα and C/EBPβ were up-regulated (Figure 5G). Overall, these results suggested that PTPN6 is decisive to the beneficial effect of METTL14 on BMSCs from SONFH patients.

### PTPN6-mediated METTL14 activated the Wnt signaling pathway

**Figure 6:**
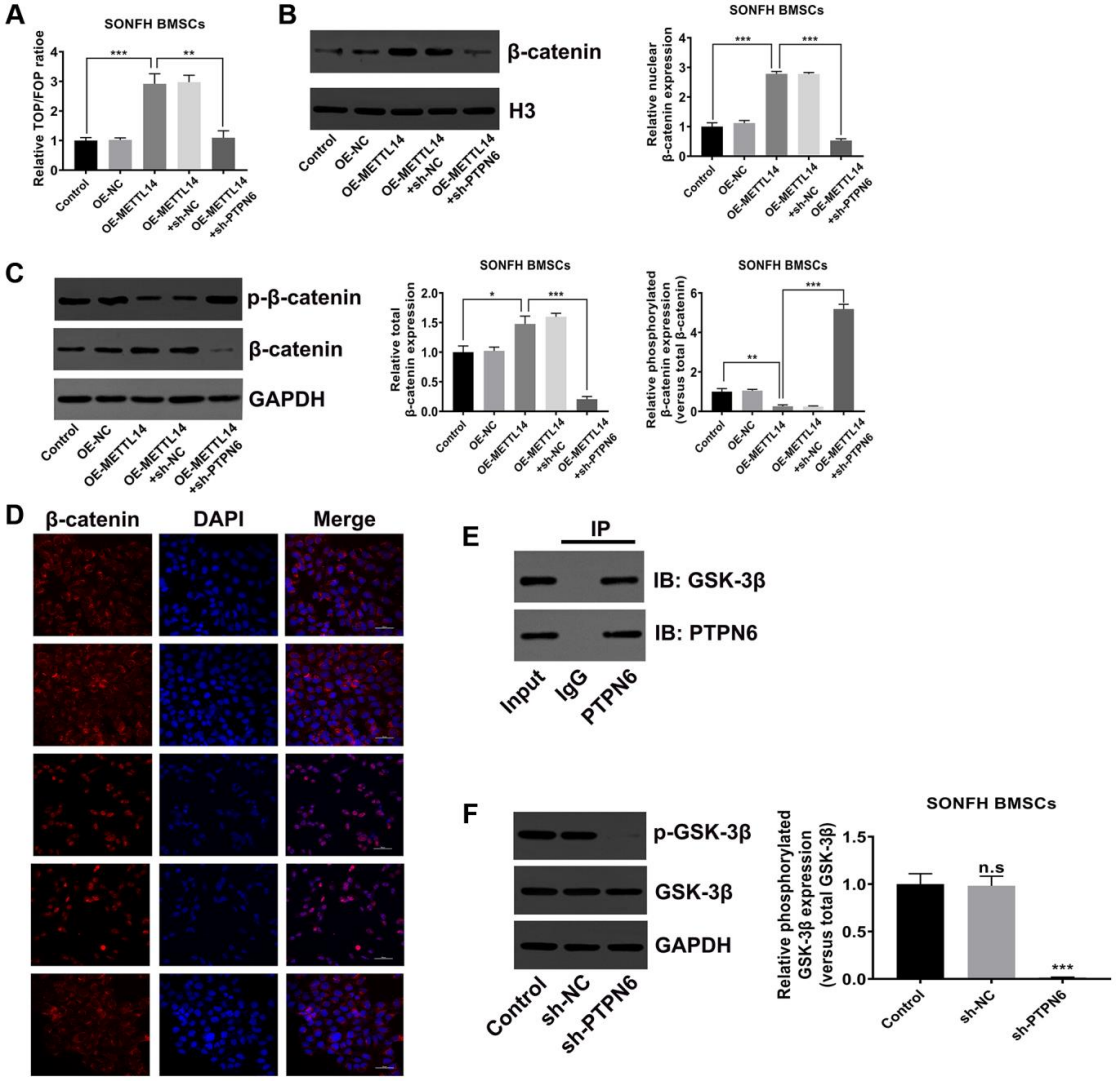
**PTPN6-mediated METTL14 activated the Wnt signaling pathway.** (**A**) The activity of Wnt signaling in SONFH BMSCs was analyzed by TOP/FOP-Flash reporter assay. (**B**) The protein level of β-catenin in the nucleus was measured by western blot. (**C**) Western blot detected the total and phosphorylated β-catenin levels in SONFH BMSCs treated as indicated. (**D**) Immunofluorescence staining was used to analyze the distribution of β-catenin in the cytoplasm and nucleus after METTL14 overexpression and PTPN6 knockdown. Scale bar = 50 μm. (**E**) The interaction of PTPN6 and GSK-3β in BMSCs was verified by co-IP assay. (**F**) The effect of PTPN6 inhibition on the phosphorylation level of GSK-3β in SONFH BMSCs was determined by western blot. ^*^P < 0.05, ^**^P < 0.01, ^***^P < 0.001, n.s is no significance.

The canonical Wnt signaling pathway is the essential signal transduction involved in the osteogenic differentiation of stem cells [35–37]. To verify whether METTL14 affects the activity of the Wnt signaling pathway in SONFH BMSCs, we initially conducted TOP/FOP-Flash reporter assay. The results showed that the activity of the Wnt signaling pathway was heightened in METTL14 overexpressed cells, and PTPN6 knockdown reversed this effect (Figure 6A). The western blot indicated that METTL14 overexpression resulted in the up-regulation of total and nuclear β-catenin and the decrease of phosphorylated β-catenin, while PTPN6 knockdown diminished the abundance of β-catenin in total protein and nucleoprotein, and the phosphorylation of β-catenin protein was augmented simultaneously (Figure 6B, 6C). Moreover, the immunofluorescence staining showed lower level of β-catenin in the nuclei of the control group than METTL14 overexpression group. The up-regulation of METTL14 obviously induced the translocation of β-catenin (red) into the nucleus (blue). PTPN6 knockdown evidently attenuated the effect of METTL14, as PTPN6 knockdown almost completely blocked the nuclear translocation of β-catenin (Figure 6D). GSK-3β assumes an essential role in the degradation of β-catenin. According to co-IP experiment, we found that PTPN6 could interact with GSK-3β in BMSC (Figure 6E). In addition, in SONFH BMSCs, PTPN6 inhibition obviously reduced the phosphorylation level of GSK-3β (Figure 6F). In conclusion, PTPN6-mediated METTL14 activates the Wnt signaling pathway in BMSCs from SONFH patients.

## DISCUSSION

Glucocorticoids are widely used to clinically treat autoimmune diseases and inflammatory-dependent diseases. Therefore, the incidence of femoral head necrosis caused by high-dose glucocorticoids is gradually increasing. With the in-depth study of the pathogenesis of SONFH, the role of BMSCs has attracted wide attention [38]. The previous study found that in the animal model of SONFH, there was a large amount of lipid deposition in the proximal femur and femoral head [13], and the osteogenic ability of BMSCs was inhibited [12, 14], which suggested that the bone repair defect caused by imbalanced osteogenic/adipogenic differentiation of BMSCs may be an important characteristic of SONFH.

m6A RNA modification is the most abundant endogenous RNA modification pattern, which widely exists in mRNA and non-coding RNA in eukaryotic cells [21]. Recent studies have found that m6A modification is dynamic and reversible in different developmental stages, tissues and physiological processes [19, 39]. Similar to DNA methylation or histone methylation, m6A RNA methylation is also catalyzed by methyltransferase and demethylase systems [17]. As the main component of the m6A methyltransferase complex, METTL14 is involved in the progression of multiple diseases in an m6A dependent manner. Weng et al. demonstrated that METTL14 inhibits hematopoietic progenitor differentiation and promotes leukemogenesis via m6A modification [40]. Ma et al. revealed that METTL14 suppresses the metastatic potential of hepatocellular carcinoma by modulating primary microRNA-126 process in m6A dependent manner [41]. However, the role of m6A modification in SONFH has not been reported. In this study, we found that the expression level of METTL14 was down-regulated in necrotic bone tissues and SONFH BMSCs, which resulted in a decrease of the m6A level. Moreover, overexpression of METTL14 in BMSCs from SONFH patients not only up-regulated the level of m6A modification in total RNA, but also enhanced cell proliferation and osteogenic differentiation, and simultaneously inhibited adipogenic differentiation.

PTPN6 is a protein tyrosine phosphatase located in the cytoplasm and participates in the regulation of immune function, tissue inflammation, cell proliferation and other various pathological processes [42–44]. Jiang et al. found that SHP1 (alias of PTPN6) knockout mice were susceptible to osteoporosis due to SHP1 could bind to GSK-3β and inhibit its kinase activity, thus activating the Wnt signaling pathway [45]. As an evolutionarily conserved signaling pathway, the canonical Wnt signaling pathway is crucial for renewal, proliferation, and differentiation of stem cells during bone development and tissue homeostasis [46, 47]. When the Wnt receptor complex is activated, GSK-3β is triggered to dissociate from the APC/Axin/GSK-3β complex, and the stable β-catenin is transported into the nucleus, where it binds to the LEF/TCF transcription factor and promotes the downstream gene transcription of this pathway [48, 49]. Accumulating evidence suggests that glucocorticoids inactivate Wnt signaling through different mechanisms and further affect the osteogenic and adipogenic differentiation of BMSCs [9, 50–52]. In this study, we identified that PTPN6 acted as the downstream target of METTL14 in BMSCs by performing mRNA sequencing. METTL14 enhanced the stability of PTPN6 mRNA in an m6A-dependent manner, thus promoting the expression of PTPN6. Furthermore, METTL14 promoted the translocation of β-catenin into the nucleus through the interaction of PTPN6 and GSK-3β, and ultimately accelerated the activation of Wnt signal transduction.

In summary, our results demonstrated that the m6A methyltransferase METTL14 can promote proliferation and osteogenic differentiation of SONFH BMSCs by up-regulating the m6A level of PTPN6 and activating the Wnt signaling pathway. These findings suggest that the METTL14-PTPN6 axis may be a potential target for the treatment of SONFH.

## MATERIALS AND METHODS

### Patients

From January 2019 to December 2019, 20 patients with steroid-associated osteonecrosis of the femoral head who underwent hip arthroplasty at the Department of Orthopedics of Henan Provincial People’s Hospital were selected as the SONFH group and 20 patients with femoral neck fracture who underwent hip arthroplasty served as the control group. Bone marrow and femoral head tissues were collected during the operation. Table 1 summarizes the clinical characteristics of all patients. The present study was performed in accordance with the ethical standards as laid down in the 1964 Declaration of Helsinki and its later amendments or comparable ethical standards, and this study was approved by the Ethics Committee of Henan Provincial People’s Hospital (Approval number: 2017237). All patients signed informed consent before operation.

**Table 1:** Clinical characteristics of patients.

	**SONFH (*n* = 20)**	**Control (*n* = 20)**	***p*-value**
Age (years)	49.8 ± 7.6	52.1 ± 8.4	0.22
Gender (Male/Female)	11/9	10/10	0.77
Body Mass Index (kg/m^2^)	26.1 ± 3.2	24.3 ± 2.7	0.18
Glucocorticoid Medication	20 (100%)	1 (5%)	<0.001
Total Cholesterol (mmol/L)	6.23 ± 0.85	4.68 ± 0.72	<0.01

### Bone marrow separation and cell culture

Five milliliters of bone marrow were procured from the proximal femur during hip arthroplasty. BMSCs were isolated and cultured as described in previous study [12]. Briefly, mononuclear cells in the bone marrow were centrifuged with an equal volume of 1.073 g/ml Percoll solution (Sigma, USA) at 2000 rpm for 30 minutes. Then, cells were collected, suspended in DMEM/F12 medium (HyClone, USA) containing 10% fetal bovine serum (Gibco, USA) and 1% penicillin-streptomycin (Biosharp, China), and cultured at 37°C, 5% CO_2_ and 99% relative humidity. In order to maintain the consistent biological characteristics, experiments were carried out when the cells were subcultured to the second generation and were no longer used after they were subcultured to the fifth passage.

### Flow cytometry

In order to verify that cells we extracted were BMSCs, we detected the expression of cell surface markers by flow cytometry. Briefly, we collected cells at the second passage and resuspended them in PBS. Then, 2 × 10^5^ cells were incubated with PE or FITC labeled cell surface marker antibodies against CD29, CD34, CD44 and CD45 (BD Biosciences, USA), as well as isotype control antibody at 4°C for 30 minutes in the dark. After the incubation is completed, the samples were analyzed by the flow cytometer (BD Biosciences, USA).

### Cell transfection

The METTL14 overexpression plasmid (termed OE-METTL14) and the negative control (termed OE-NC) were purchased from OBIO Technology (Shanghai, China). Short hairpin RNA targeting PTPN6 (termed sh-PTPN6) and the negative control (termed sh-NC) were designed by GeneChem (Shanghai, China) and cloned into GV112 lentiviral vectors (GeneChem, Shanghai). The cells were infected with the viruses in the presence of Polybrene for 48 hours. Then, positively transfected cells were selected for 7–10 days using 2 μg/ml puromycin. The overexpression and knockdown efficiencies were determined by qRT-PCR and western blot assay.

### Osteogenic and adipogenic differentiation

For osteogenic differentiation, BMSCs were cultured in complete DMEM/F12 medium containing 0.25 mM ascorbic acid, 10 mM β-glycerophosphate and 10 nM dexamethasone for 21 days.

Adipogenic differentiation of human BMSCs was performed according to an earlier study [53]. Cells were cultured in adipogenesis-inducing medium: complete DMEM/F12 medium containing 1 μM dexamethasone, 0.2 mM indomethacin, 0.5 mM isobutyl-methylxanthine and 0.01 mg/ml insulin. Three days later, the adipogenesis-inducing medium was changed to adipogenesis maintenance medium (complete DMEM/F12 medium containing 0.01 mg/ml insulin). After 24 hours, switched back to adipogenesis-inducing medium. The cells were alternately cultured in the two media for 3 cycles, after which the cells were maintained in the maintenance medium for one week.

### Alizarin red S staining and oil red O staining

The cells induced by osteogenic differentiation or adipogenic differentiation were fixed with polyformaldehyde for 30 minutes and then stained with 40 nM alizarin red solution (pH 4.2) or filtered oil red O solution at room temperature for 30 minutes. The nonspecific staining was removed with PBS. Stained BMSCs were observed and photographed by an inverted microscope (Olympus, Japan).

### Alkaline phosphatase (ALP) activity assay

ALP activity assay was performed with the Alkaline Phosphatase Assay Kit (Abcam, UK). Initially, 1% Triton X-100 was added to lyse cells for 20 minutes, followed by centrifugation at 10000 g for 5 minutes, and the supernatant was collected. Thereafter, the reaction mixture was added and incubated at room temperature for 60 minutes. Finally, the assay buffer was added and incubated again for 30 minutes, the absorbance at 450 nm was measured by a microplate reader (Thermo Scientific, USA).

### Cell proliferation assay

Cell Counting Kit-8 (CCK-8, Beyotime, China) was used to measure the cell proliferation activity according to the instructions. In brief, the cells were cultured in 96-well plates for 0, 24, 48 and 72 hours and then exchanged for 100 μl serum-free medium, and 10 μl CCK-8 solution was added to each well. The absorbance at 450 nm was measured by a microplate reader after incubation at 37°C for 2 hours.

### EdU assay

According to the precedent study described [54], the EdU assay kit (RiboBio, China) was adopted to determine the cell proliferation ability. Cells were seeded into 96-well plates at a density of 5 × 10^5^ cells per well, and cultured under standard conditions for 72 hours. Then, they were incubated with EdU buffer at 37°C for 2 hours, fixed with 4% formaldehyde for 30 minutes and permeabilized with 0.1% Triton X-100 for 20 minutes. EdU solution was added to the culture, followed by the staining of nuclei with DAPI. The results were visualized by a fluorescence microscope (Olympus, Japan).

### Quantitative real-time PCR

Total RNA in femoral head tissues and BMSCs was extracted using TRIzol Reagent and used to synthesize cDNA with the One-Step RT-PCR Kit (Thermo Scientific, USA). qRT-PCR was performed using the SYBR Primer-Script RT-PCR Kit (TaKaRa, Japan) with a CFX96 Touch quantitative PCR system (Bio-Rad, USA). Primers were generated by Sangon Biotechnology (Shanghai, China), and the primer sequences were as follows: METTL14, 5′-AGAAACTTGCAGGGCTTCCT-3′ (forward) and 5′-TCTTCTTCATATGGCAAATTTTCTT-3′ (reverse); PTPN6, 5′-GCCTGGACTGTGACATTGAC-3′ (forward) and 5′-ATGTTCCCGTACTCCGACTC-3′ (reverse); METTL3, 5′-CAAGCTGCACTTCAGAC GAA-3′ (forward) and 5′-GCTTGGCGTGTGGT CTTT-3′ (reverse); WTAP, 5′-ACGCAGGGAGAAC ATTCTTG-3′ (forward) and 5′-CACACTCGGCTGC TGAACT-3′ (reverse); FTO, 5′-TTCATGCTGGAT GACCTCAATG-3′ (forward) and 5′-GCCAACTGAC AGCGTTCTAAG-3′ (reverse); ALKBH5, 5′-TCACT GCATACGGCCTCAGGACAT-3′ (forward) and 5′-T TAGAGCAGGGTCCCTGTTGT-3′ (reverse); GAPDH, 5′-TGGTATCGTGGAAGGACTCA-3′ (forward) and 5′-CCAGTAGAGGCAGGGATGAT-3′ (reverse).

### Western blot

Total protein was extracted with RIPA lysate containing cocktail protease inhibitor (Beyotime, China), and nuclear protein was extracted by the CelLytic NuClear Extraction Kit (Sigma, USA). The protein concentration was detected with the BCA Protein Assay Kit (Beyotime, China). Protein lysates were separated by 12% SDS-PAGE and transferred onto PVDF membranes. Then, PVDF membranes were blocked with 5% nonfat milk and incubated with primary antibodies. The following primary antibodies were used in this study: anti-METTL14 (ab252562, Abcam), anti-PTPN6 (ab227503, Abcam), anti-RUNX2 (ab76956, Abcam), anti-PPARγ (ab59256, Abcam), anti-C/EBPα (ab40764, Abcam), anti-C/EBPβ (ab15049, Abcam), anti-β-catenin (ab6302, Abcam), anti-p-β-catenin (ab11350, Abcam), anti-GSK-3β (ab32391, Abcam), anti-p-GSK-3β (ab68476, Abcam), anti-GAPDH (ab8245, Abcam), anti-Histone H3 (ab1791, Abcam). GAPDH and H3 were used as the internal controls for total protein and nuclear protein, respectively. Horseradish peroxidase-conjugated secondary antibody was used to develop the blots. Blots were detected with the ECL Western Blotting Substrate Kit (ab65623, Abcam) on an ECL system (Bio-Rad, USA).

### m6A RNA methylation quantification

The content of m6A in total RNA was measured by the m6A RNA Methylation Assay Kit (ab185912, Abcam). Briefly, isolated total RNA and bound RNA to the assay wells, and the wells were washed and incubated with capture antibody. After that, rewashed wells and added detection antibody and enhancer solution. Finally, the color developing solution was added and measured the absorbance at 450 nm by a microplate reader.

### mRNA sequencing

The high-quality total RNA was extracted using TRIzol reagents from 1 × 10^6^ METTL14-overexpressing cells and control cells per duplicate and genomic DNA removed using a DNA-free Kit (ThermoFisher, USA). Library construction was conducted using a Collibri 3′ mRNA Library Preparation Kit (ThermoFisher, USA) according to the manufacturer’s instructions. Quality control and sequencing were performed by Genenergy Bio-Technology Inc. The DEGseq software package was used to identify differentially expressed genes between the METTL14 overexpression group and the control group with parameters *p*-value <0.05 and false discovery rate <0.05.

### MeRIP-qPCR

The m6A level of the individual gene was determined by MeRIP-qPCR assay. According to the previous study [41], purified poly (A) RNA from total RNA was obtained using the Dynabeads mRNA Purification Kit (Thermo Scientific, USA). A/G immunomagnetic beads were incubated with anti-m6A antibody (ab208577, Abcam) or anti-IgG antibody (ab172730, Abcam) in the immunoprecipitation buffer to immunoprecipitate m6A-RNA complexes. Thereafter, the complexes were digested by Proteinase K (20 mg/ml), and the methylated mRNA of PTPN6 was measured by qRT-PCR.

### RNA stability

To evaluate the effect of METTL14 on the stability of PTPN6 mRNA, we detected the half-life of PTPN6 mRNA. Actinomycin D (5 μg/ml) was used to intervene in OE-NC and OE-METTL14 transfected BMSCs from SONFH patients, and total RNA was extracted at 0, 2, 4, 6 and 8 hours. The level of PTPN6 mRNA was measured by qRT-PCR, and the half-life was obtained by nonlinear regression analysis.

### Luciferase reporter assay

Luciferase reporters were generated by cloning wild type 3′UTR of PTPN6 mRNA or mutant type 3′UTR of PTPN6 mRNA (mutant A to T at position 859) into psiCHECK2 (Promega, USA). Then, cells were seeded in 24-well plates and co-transfected with 150 ng of renilla reporters and OE-METTL14. 48 hours later, collected cells and luciferase activities were detected using the Dual-Luciferase Reporter Assay System (Promega, USA) according to manufacturer’s protocol.

### TOP/FOP-Flash reporter assay

TOP/FOP-Flash reporter assays were used to determine the transcriptional activity of the Wnt/β-catenin pathway. BMSCs were seeded in 24-well plates and transfected with the TOP/FOP-Flash reporter (Biovector NTCC Inc., China). After 48 hours, the luciferase activity was measured with the Dual-Luciferase Reporter Assay System (Promega, USA). The luciferase activity of each sample was normalized to the respective Renilla luciferase activity.

### Immunofluorescence staining

Cells cultured on cover slips were washed twice with cold PBS and fixed with paraformaldehyde for 15 minutes at room temperature and were permeabilized with 0.1% Triton X for 20 minutes. Cells were incubated with 1% bovine serum albumin for 40 minutes to block the nonspecific antibody binding sites and subsequently incubated with anti-β-catenin antibodies (1:200) overnight at 4°C. Afterwards, the cells were washed three times with PBS and incubated with a 1:500 dilution of an APC-conjugated secondary antibody for 1 hour. Finally, cells were stained with DAPI to visualize the nuclei. Images were captured by a fluorescence microscope.

### IHC staining

The femoral head tissues were decalcified at room temperature in 15% EDTA for 5 weeks. After decalcification, the sample was embedded in paraffin. Four micrometer paraffin slides were prepared on glass slides coated with polylysine and then dried for one hour at 60°C. The slides were blocked with 5% BSA and then stained with anti-METTL14 antibody (1:100) or anti-PTPN6 antibody (1:100) at 4°C overnight. Finally, the slides were incubated with HRP-labeled secondary antibody. The slides were visualized by an inverted microscope.

### Co-immunoprecipitation assay

Total protein lysates for immunoprecipitation assay were extracted with IP-lysis buffer (ThermoFisher, USA). The supernatant was collected by centrifugation at 12000 rpm for 10 minutes at 4°C. For immunoprecipitation, the supernatant was mixed with anti-PTPN6 antibody (ab227503, Abcam) or anti-IgG antibody (ab172730, Abcam) and 50 μL of protein A/G Dynabeads (ThermoFisher, USA) at 4°C overnight. After that, the beads were washed three times with pre-cooled IP lysis buffer, and immunoprecipitates were subjected to western blotting.

### Statistical analysis

All experiments were independently repeated at least three times. Data in this study are expressed as the mean ± SD. GraphPad Prism 7.0 software was used for all data analysis. The difference between two groups was analyzed by *t*-test. One-way analysis of variance (ANOVA) was used for comparison among the multiple groups. The difference of clinical features between SONFH group and control group was analyzed via χ^2^ analysis. The linear association between METTL14 and PTPN6 was tested by Pearson’s Correlation Coefficient. *P*-value <0.05 was considered statistically significant.
